# Major destructive asymptomatic lumbar Charcot lesion treated with three column resection and short segment reconstruction. Case report, treatment strategy and review of literature

**DOI:** 10.1051/sicotj/2017056

**Published:** 2017-12-11

**Authors:** Kestutis Valancius, Gaurav Garg, Madalina Duicu, Ebbe Stender Hansen, Cody Bunger

**Affiliations:** Spine Surgery Unit, Department of Orthopaedic Surgery, Aarhus University Hospital, Noerrebrogade 44, 8000 Aarhus C Denmark

**Keywords:** Charcot’s spine, Asymptomatic, Lumbar, Three column resection

## Abstract

Charcot's spine is a long-term complication of spinal cord injury. The lesion is often localized at the caudal end of long fusion constructs and distal to the level of paraplegia. However, cases are rare and the literature relevant to the management of Charcot's arthropathy is limited. This paper reviews the clinical features, diagnosis, and surgical management of post-traumatic spinal neuroarthropathy in the current literature. We present a rare case of adjacent level Charcot's lesion of the lumbar spine in a paraplegic patient, primarily treated for traumatic spinal cord lesion 39 years before current surgery. We have performed end-to-end apposition of bone after 3 column resection of the lesion, 3D correction of the deformity, and posterior instrumentation using a four-rod construct. Although the natural course of the disease remains unclear, surgery is always favorable and remains the primary treatment modality. Posterior long-segment spinal fusion with a four-rod construct is the mainstay of treatment to prevent further morbidity. Our technique eliminated the need for more extensive anterior surgery while preserving distal motion

## Introduction

Charcot's spine or spinal neuroarthropathic disease is a relatively bizarre, ongoing catastrophic activity affecting the osseous and ligamentous structures of the spine. It is often seen in the setting of a pre-existing condition characterized by decreased afferent innervation sufficiently severe to impair the normal protective sensation of the joints supporting the vertebral column [1]. Due to lack of symptoms early in the disease process, prevalence of Charcot's spine remains unclear. However, some reports suggest 6–21% of spinal involvement in patients suffering from peripheral neuroarthropathy [2,3]. Due to an asymptomatic evolutionary nature of the disease, most patients will progress to advanced stages of destruction before diagnosis is established.

Charcot's lesion is often seen in association with other pathologies, however; the most contemporary literature comprises patients with traumatic spinal cord injury [4,5]. The overlap of imaging features with those of other etiologies, adds up to a diagnostic dilemma [6]. Long-term immobilization with body cast/torso has been practiced in the past but is associated with high failure rates [7]. The trend has changed towards a treatment paradigm of primary surgical intervention. The goal of surgery is restoration of normal sagittal balance and aggressive debridement of the entire region of denervated sclerotic bone. This is typically achieved by 3 column resection and 3D reconstruction [8]. Our objective is to report an unusual case of adjacent level Charcot's neuroarthropathy of the lumbar spine as a late complication of traumatic spinal cord injury, treated by 3 column vertebral resection and fusion, spinal instrumentation as a one-stage procedure.

## Case report

A 53-year-old male, wheelchair user, presented to our department with 3 years history of clunking noise in his back while transferring, shortened torso and deteriorated sitting balance. The patient suffered complete paraplegia at T10 from road a traffic accident 39 years back. Primarily the patient was treated by performing T8–T12 fusion with Harrington rod fixation. Spinal instrumentation was removed 10 years later due to implant prominence and pain in upper back, which was completely relieved after implant removal. Despite the patient was wheelchair-user, he managed independently to do his routine self-care activities, completed education and work as a leader of his own international company.

The patient was referred to our clinic due to new difficulties to reach shelf's in his kitchen while sitting in the wheelchair, tumour prominences and clunking noise in his back. Clinical examination revealed a kypho-scoliosis with instability distal to the thoracic-lumbar junction. There was a 15 cm increase in body length from sitting to supine posture. He had complete flaccid paralysis of both lower limbs, with sensory loss below T9, and no contractures. He had no history of fever or weight loss. No recent changes had occurred in relation to his bowel and bladder habits.

**Figure 1:**
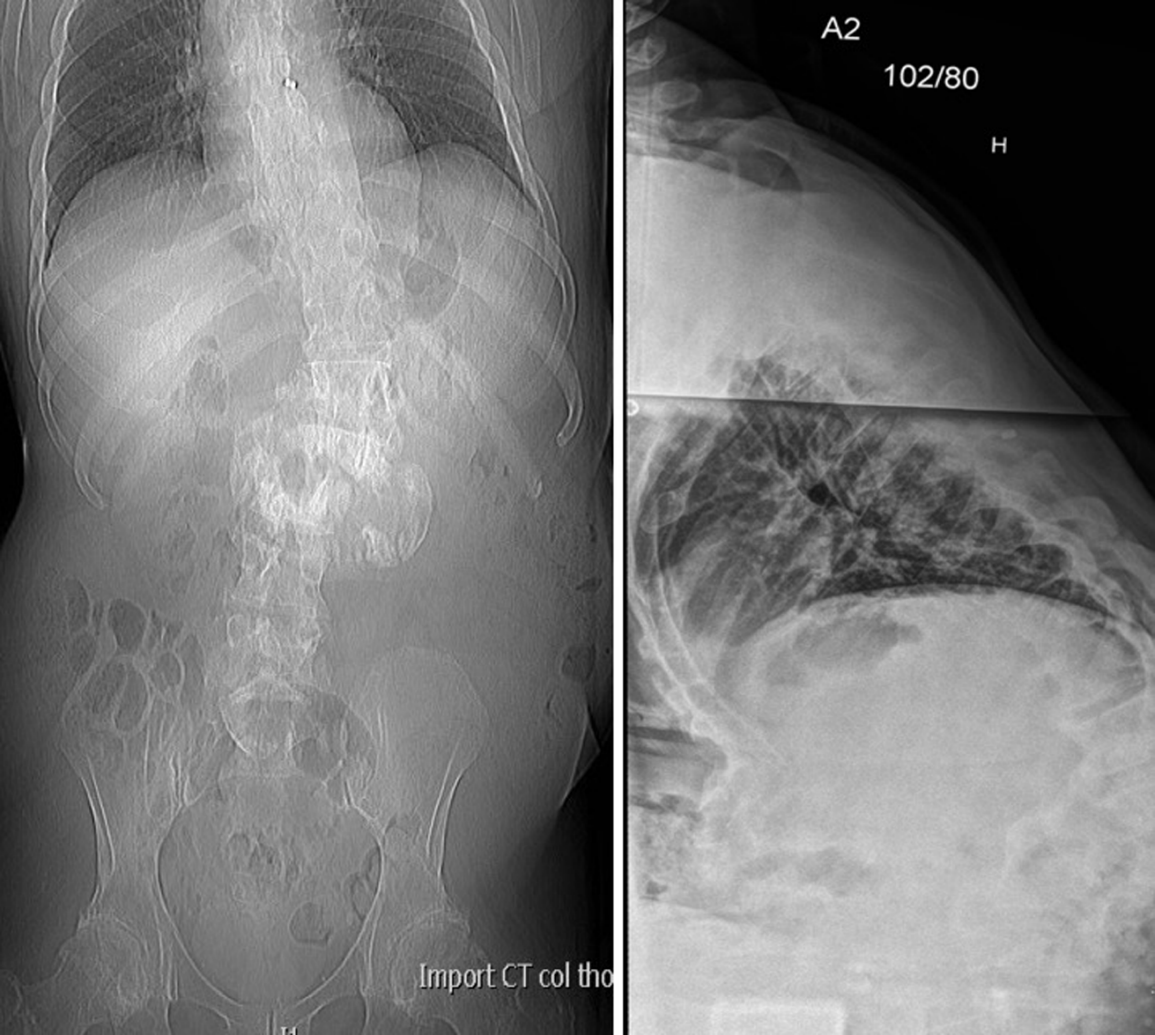
Radiographs showing marked destruction of L2 and L3 vertebrae with paravertebral hypertrophic ossification and kypho-scoliotic deformity.

**Figure 2:**
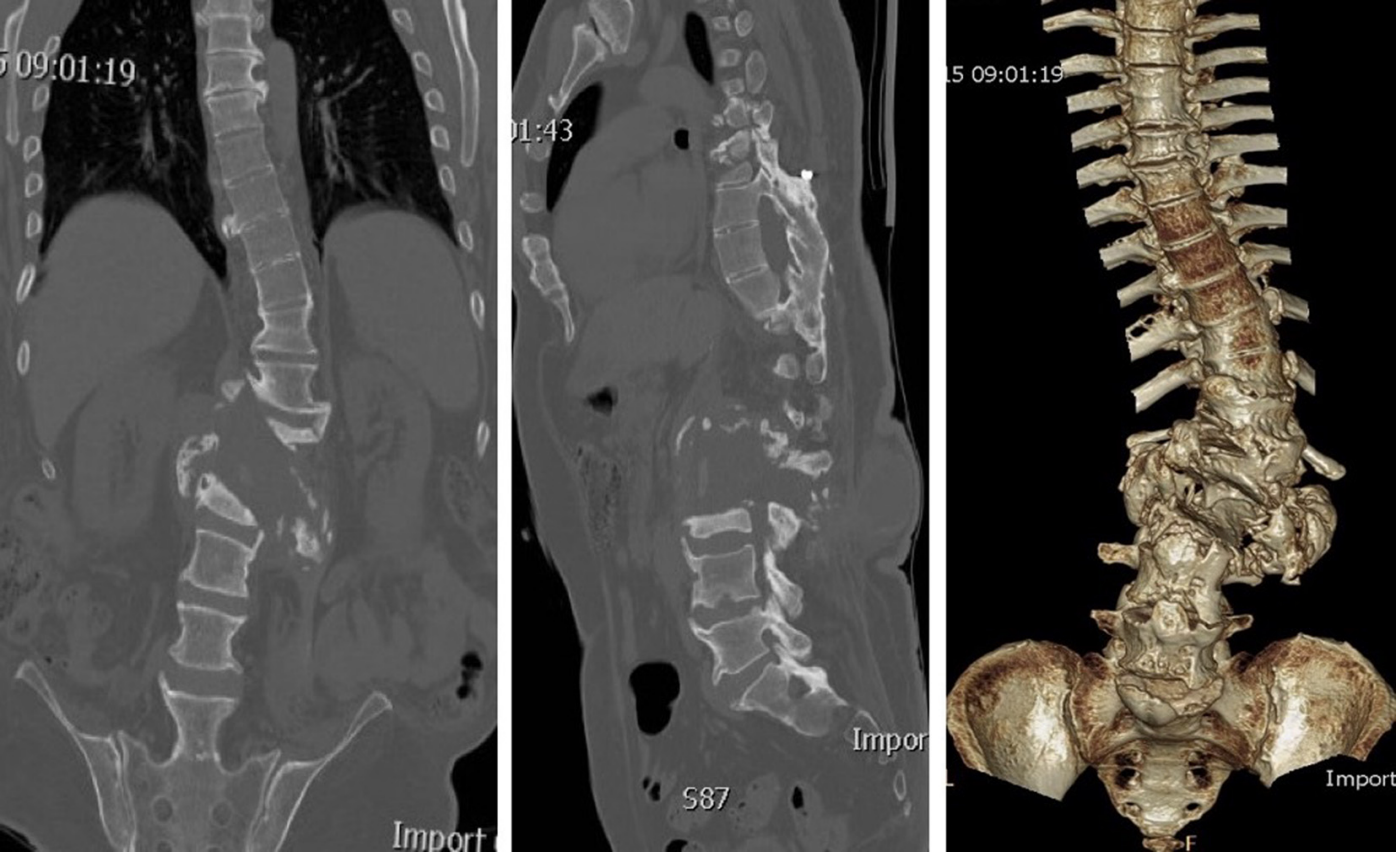
CT scan at presentation showing bony destruction and para-vertebral new bone formation.

**Figure 3:**
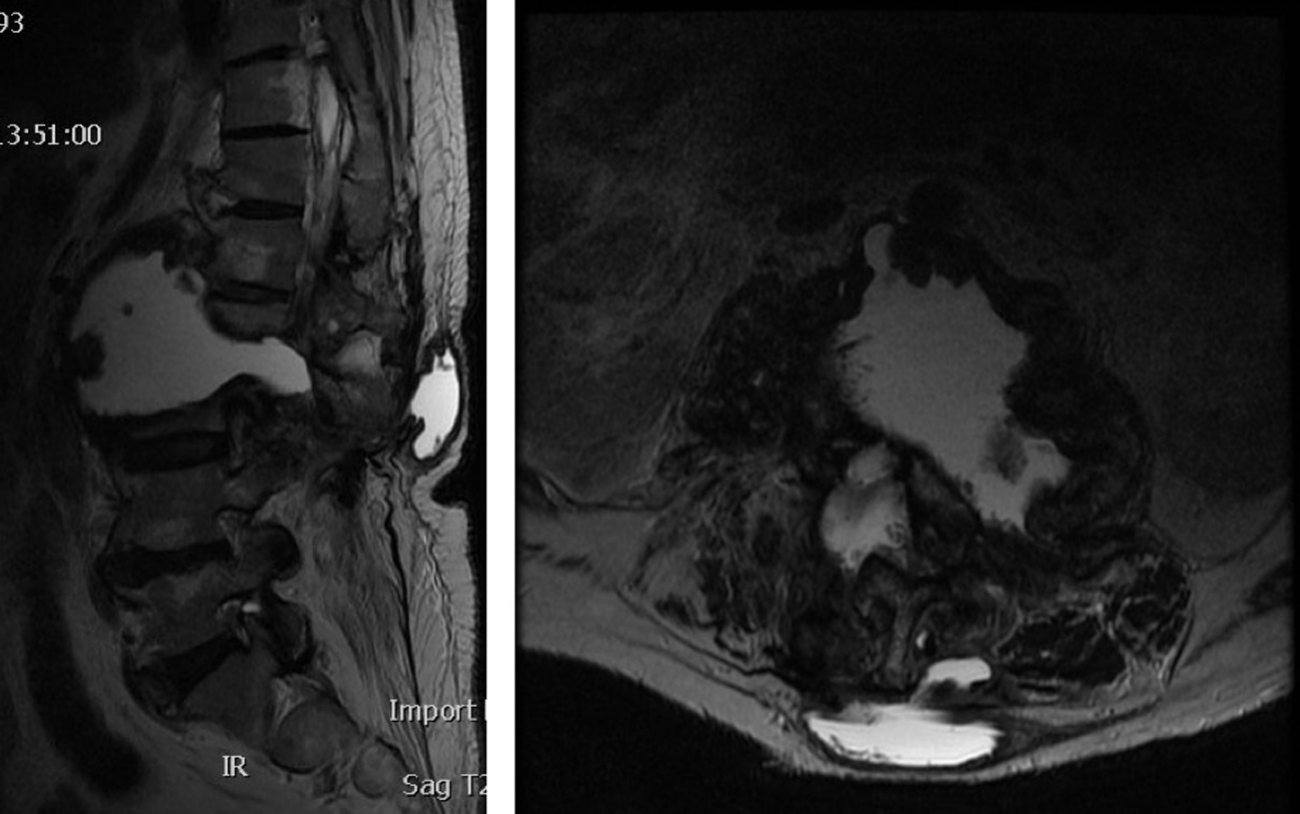
Pre-operative MRI in supine position showing large fluid filled cavitary lesion between L2 and L3 vertebrae extending to the posterior elements and associated bony resorption.

Radiographs showed gross destruction of L2 and L3 vertebral bodies leading to kypho-scoliosis at the upper lumbar region with a Cobb angle of 70 degrees in the sagittal plane, paravertebral hypertrophic ossification involving an area from T12 to L3, with destruction of posterior elements, and lateral translation of L2 over L3 (Figure 1). A CT scan confirmed partial resorption of upper lumbar vertebral bodies and gross para-spinal ossification and deformity (Figure 2). MRI demonstrated the complex vertebral body destruction, peripheral bony debris, and paraspinal mass with a huge fluid filled cavity extending into posterior elements. A minor syrinx was also seen at T10/T11 following previous spinal cord injury (Figure 3). Laboratory analysis reveals no signs of infectious pathology. Based on clinical history, imaging, and laboratory findings, the diagnosis of Charcot's lesion was made. Due to the massive cavitary lesion and hyper-intense signal in T2 weighted images, angiography was performed to rule out hyper-vascular nature of the lesion and in order to perform pre-operative embolization.

**Figure 4:**
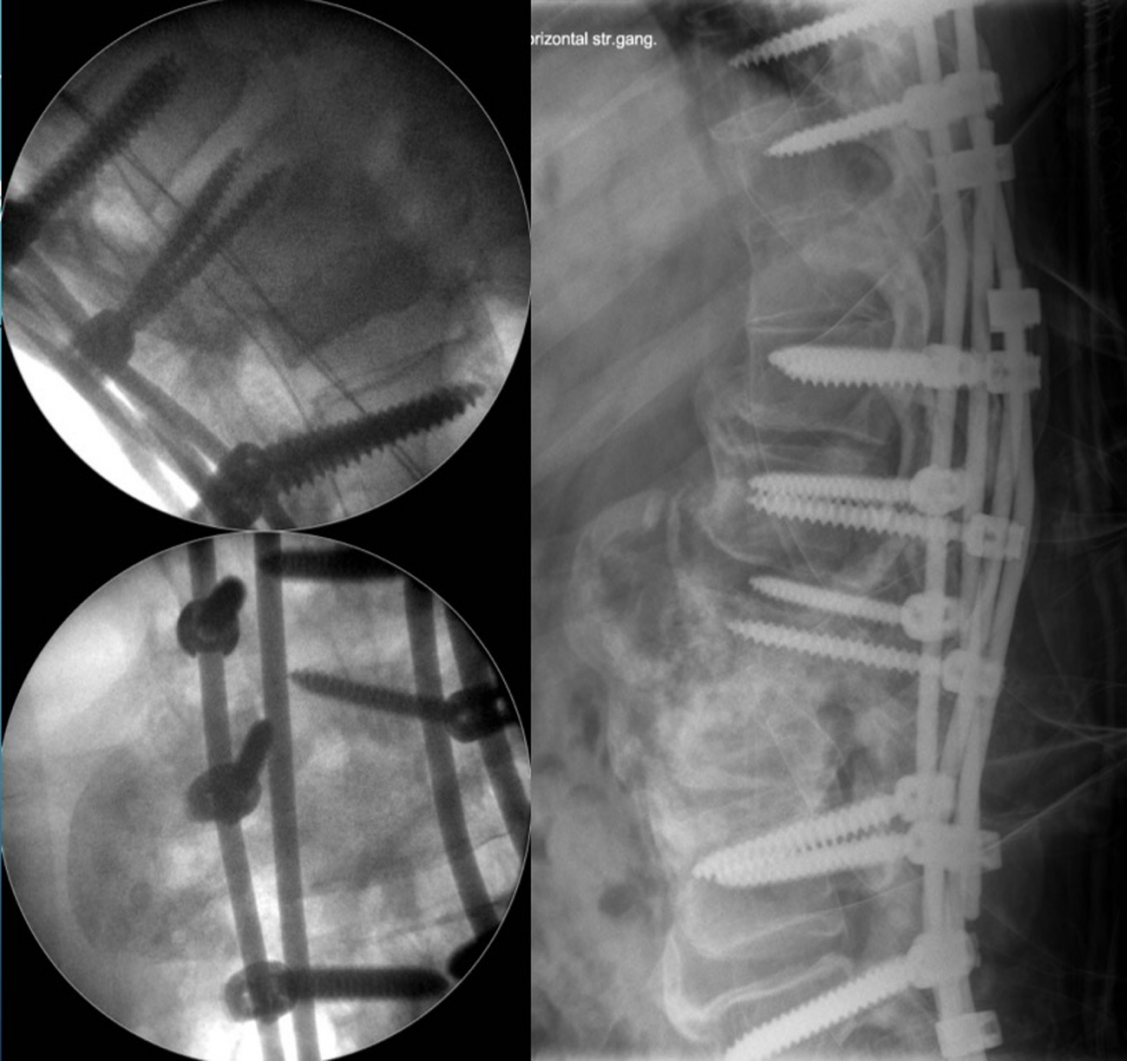
Intra-operative fluoroscopy image showing final fixation with four rod construct and immediate post operative radiograph showing good 3D correction of the deformity with stable fixation.

Goals of surgical treatment comprised complete excision of the lesion, correction of the deformity and stabilization of the spine. Exposure of the affected level through the posterior approach showed complete destruction of posterior elements and a pseudo joint cavity filled with yellowish coloured fluid without relation to the spinal canal. Tissue samples were taken from neuropathic segment for culture and histological assessment. Pedicle screws were inserted in T12–L1 and L4–L5, unilateral rod was used for temporary stabilization and distraction to work upon the lesion. Complete 3 column resection of the pseudoarthrotic cavity and curettage of sclerotic avascular endplate bone was performed. Resection was kept confined to the anterior pseudocapsule of the lesion. The spine was realigned and Ends of the resected cavity were approximated and temporarily stabilized. A structural allograft (femoral head from the bone bank) was placed in the created void. The rest of the space was filled with morselized local graft. There was good bone quality in the uninvolved vertebrae felt during screw insertion. Intra-operative fluoroscopy revealed kyphosis in thoraco-lumbar region even after closure of the resection site. The construct was extended to T9 proximally, and bilateral Smith Peterson osteotomy was performed between T10/T11 and T11/T12. Correction of the deformity was achieved and the rods were in situ bended and placed. A four rod construct was used for the fixation to prevent future risk of rod breakage (Figure 4). The lateral posterior elements were decorticated and packed with morselized allograft. Blood loss was 1.5 L in the 6 h procedure.

**Figure 5:**
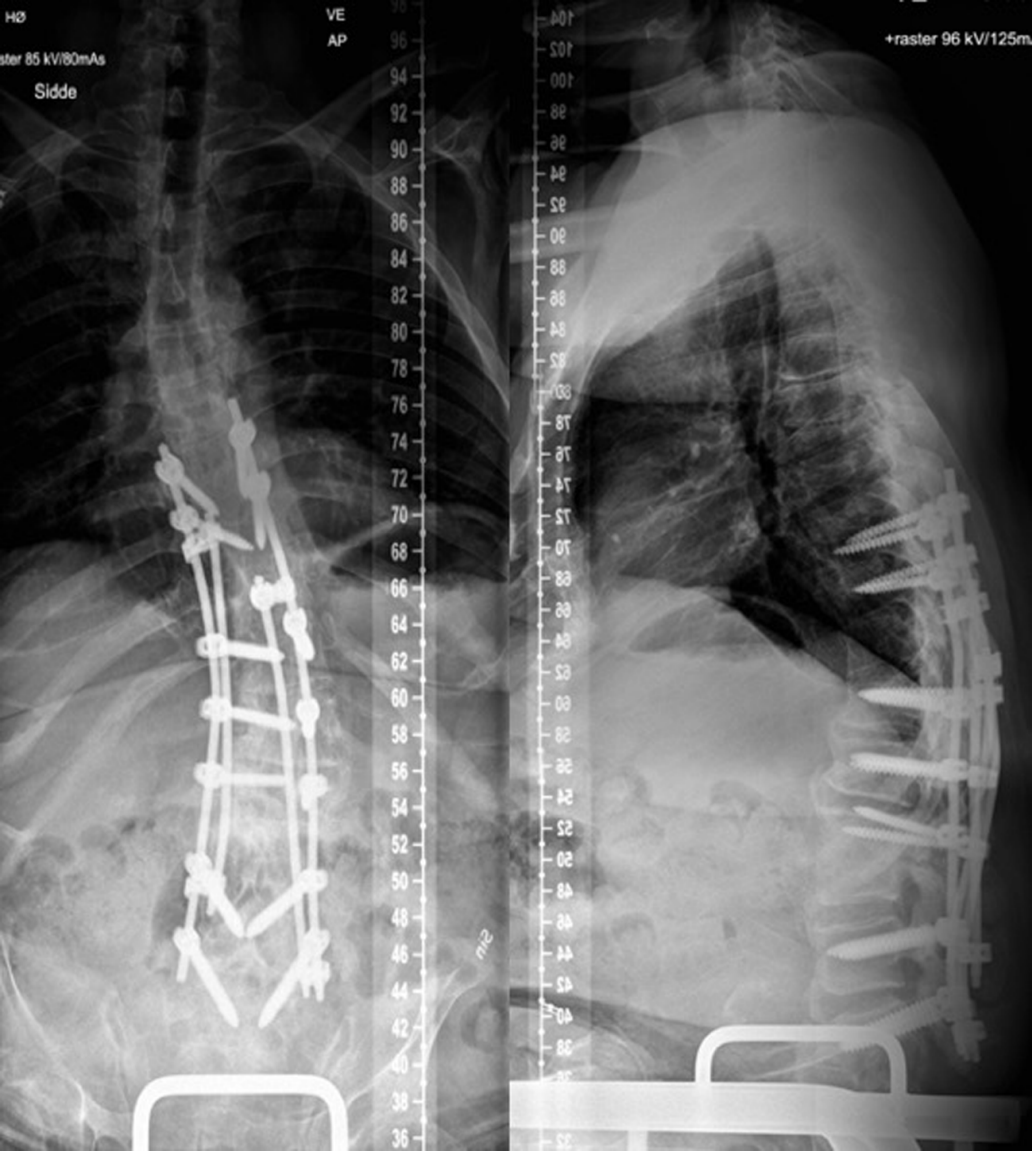
1 year follow up radiograph shows good consolidation at the pseudoarthrosis site and correction of the deformity.

Post-operatively the patient recovered uneventfully except for a single episode of reduced blood pressure, for which he stayed an additional day in intensive care. There was 2.5 cm shortening in the posterior column compared to the pre-operative length. He was allowed to mobilize in wheelchair without brace immediately after surgery. Clinically the patient had a straight spine and reported to feel more secure and balanced while sitting in the wheelchair. Biopsy taken the wall of the cavity revealed non-specific fibrosis and the cultures were negative for any infectious pathology. The patient rehabilitated well to the pre-operative status and was allowed to transfer in and out of the wheelchair himself by 6 weeks. At the latest follow up 1 year post-operatively, the patient remained symptom-free and independent doing his routine daily activities (Figure 5).

## Discussion

Traumatic spinal cord injury was first reported as a cause of Charcot's spinal neuroarthropathy by Slabaugh in 1978 and still remains to be one of the most common etiologies. Disease is seen in association with diabetic neuropathy, tertiary syphilis, anaesthetic leprosy, syringomyelia or congenital absence of pain syndrome. Traumatic event (either repeated micro trauma or a single major event) appears to be a major aggravating factor among the plethora of diseases being described. Animal experiments demonstrated the role of trauma in the development of neuroarthropathy, since joint denervation alone was not a sufficient condition [9]. The time between the onset of neurological impairment and the development of spinal neuroarthropathy has been quite substantial averaging 17.3 years over multiple case series [10]. In our case, Charcot's spine was diagnosed after 34 years of primary injury and paraplegia. It develops due to repetitive micro-trauma in patients with lost deep pain and proprioceptive sensation, which impair the normal protective mechanisms of the intervertebral joints. In paraplegic patients transfer in or out of a wheelchair, exerts biomechanical loads in the insensate spine. Repeated micro-trauma increases joint mobility beyond normal limits, further leading to damage and starting a vicious cycle, culminating to severe instability and bone destruction. In paraplegic patients, lumbar lordosis flattens and may even progresses to kyphosis, which may exaggerate Charcot's development.

Charcot's spine is seen in patients with complete motor deficits (ASIA Grade A and B), and occurs caudal to the neurological level of injury [1]. Iatrogenic instability after decompressive laminectomies and/or transfer of excessive loads to segments adjacent to previously fused area in a paraplegic patient, has been causative factors of the disease in almost 70% cases. It has been recommended to do instrumented fusion in laminectomized paraplegic patients to prevent development of neuroarthropathy in long term [11]. Recently, Jacobs et al., reported that long spinal fusions (mean 8.4 levels) places supra physiologic loads on the adjacent segments increasing the risk of neuroarthropathy and raise a possibility of using short segmental fusions for vertebral fractures in setting of spinal cord injury [1].

Back pain is a frequent complaint in fully paralyzed patients, which may be mechanical or inflammatory in nature, and it is often difficult to differentiate it from neuropathic pain. However our patient had no pain despite the major lesion. Patients may present with sitting imbalance and audible cracking sound on transfers. Bizarre changes in neurological status like accentuated spasticity in partial hemiplegics, reduced spasticity in complete paraplegics, and autonomous dysreflexia can also be a part of disease evolution. However, appearance of painless progressive deformity is more suggestive of neuropathy. In tetraplegics or high paraplegics, dysautonomic syndromes like arterial hypertension, bradycardia, hyperhidrosis, and headache have been reported [8].

Imaging studies may be strikingly abnormal with a relative paucity of clinical symptoms. The characteristic radiographic features of Charcot's spine include enlargement of adjacent vertebrae due to dense appositional new bone formation, hypertrophic spurring involving the posterior elements, and juxtra-articulate bone disorganisation. The vacuum phenomenon within the disc space is a hallmark of spinal neuroarthropathy as it indicates preserved motion [12]. Dynamic radiographs may demonstrate excessive motion at the affected level. CT imaging may show the ossified margins of the Charcot cavity, which may lead to the suspicion of tumour. MRI demonstrated fluid collection (T1 hypo-intense and T2 hyper-intense images) around arthropathic lesion. Another differential diagnosis is chronic infectious process. Infection of a pre-existing Charcot's lesion has been reported [13]. Furthermore, ESR is non-specifically elevated in neuropathy and cannot be used to eliminate infection. In the case presented here, we have performed angiography and embolization before embarking on definitive treatment to look for any hypervascular status of the lesion and to reduce the bleeding per-operatively. The overlap of imaging features with those of other etiologies, adds up to a diagnostic dilemma and must be ruled out on clinical grounds before planning out treatment.

Immobilization is essential to halt the disease progression irrespective of stage of the disease. The trend has changed towards a treatment paradigm of primary surgical interventionin ambulatory patients and in those with incomplete paraplegia to preserve the remaining neurological function. Despite the lack of firm treatment evidence, various principles centered around the surgical treatment have evolved. However, a recent study preferred multidisciplinary conservative approach in the absence of evolving neurological compromise or infection, particularly in paraplegics or late-stage neuroarthropathies [14]. The goal of surgery is to stabilize the diseased spinal segment by an appropriate fusion technique, and to ensure radical debridement of the entire region of denervated sclerotic bone. Long fusion to pelvis for thoraco-lumbar neuroarthropathies has been advocated, especially in paraplegics involved in self transfer activities, to prevent revision surgeries for adjacent level arthropathy and hardware failure [4]. Although the risk of seeing a new Charcot's lesion developing between the instrumentation and the pelvis exists, also fusions to the sacrum create long lever arms, generating high shear forces allowing for the possibility of pseudoarthrosis [15]. Approaches for adequate debridement are subject to debate, but re-operation rates have been reported with posterior column only reconstructions. The debridement can be executed via an anterior, a postero-lateral or, a combined approach and can be performed as a single or staged procedure [16]. Thomason et al. reported good outcome with posterior surgery and eliminated the need for extensive surgery, reducing the surgical morbidity [17]. In order to address the problems related to hardware failure at the lumbo-sacral junction, Jacob et al. used a lumbo-pelvic four rod construct and BMP augmented fusion as part of a treatment regimen. They have demonstrated significant reduction in treatment failure rates, reducing the incidence of revision surgeries [1]. However, delayed surgical revision is common with re-operation rates of up to 40% [5].

## Conclusion

Traumatic spinal cord injury is a major predisposing factor to the development of Charcot's spine, which typically presents decades after the primary injury. They have high predisposition to the caudal end of long fusion constructs, distal to the level of paraplegia. Repeated micro-motion during self transferring activities in paraplegics, have high chances of developing the disease.

Although the natural course of the disease remains unclear, surgery is always favorable and remains the primary treatment modality. Posterior long-segment spinal fusion with a four-rod construct is the mainstay of treatment to prevent further morbidity. Our technique with 3 column resection and 3D correction with intravertebral allograft strut grafts in combination with maximized pedicle screws and quadruple rods presented here eliminated the need for extended fusion to the pelvis, preventing the possibility of developing pseudoarthrosis due to long lever arms. We have approached the lesion posteriorly and curetted the entire lesion, eliminating the need for most extensive anterior surgery, adding to the surgical complications and the morbidity. Quadruple rod construct used in our case, adds to the stability and prevent incidence of implant failures at bone resection site.

## Conflict of interest

The authors declare that they have no conflicts of interest in relation to this article.

## Funding

There were no sources of financial or material support for this report.

## Ethics

Project is performed in accordance with the rules of the scientific ethical committee of Central Denmark Region. Patient acceptance is warranted.
